# Sugar Metabolism in Stone Fruit: Source-Sink Relationships and Environmental and Agronomical Effects

**DOI:** 10.3389/fpls.2020.573982

**Published:** 2020-11-13

**Authors:** Rachele Falchi, Claudio Bonghi, María F. Drincovich, Franco Famiani, María V. Lara, Robert P. Walker, Giannina Vizzotto

**Affiliations:** ^1^Department of Agricultural, Food, Environmental, and Animal Sciences, University of Udine, Udine, Italy; ^2^Department of Agronomy, Food, Natural Resources, Animals and Environment, University of Padova Agripolis, Legnaro, Italy; ^3^Facultad de Ciencias Bioquímicas y Farmacéuticas, Centro de Estudios Fotosintéticos y Bioquímicos, Consejo Nacional de Investigaciones Científicas y Técnicas, Universidad Nacional de Rosario, Rosario, Argentina; ^4^Dipartimento di Scienze Agrarie, Alimentari e Ambientali, Università degli Studi di Perugia, Perugia, Italy

**Keywords:** assimilates partitioning, phloem loading/unloading, photosynthesis, water flow, fertilization, pruning, thinning, rootstock

## Abstract

The partitioning of assimilates in fruits, which are economically important sink organs, is ruled by different physiological processes and affected by both environmental and agronomical factors. The bulk of the water and solutes, required for growth, is imported into fruits and seeds through xylem and phloem. In the stone fruits, five vascular bundles enter the base of the fruit, then dividing to supply either the flesh or the seed. The main sugars accumulated in stone fruits include fructose, glucose, and sucrose, along with other minor saccharides. The mechanisms of phloem loading in these fruit species have not been fully elucidated yet, but the available data hint either an apoplastic or a symplastic type or possibly a combination of both, depending on the species and the sugar considered. Similarly, phloem unloading mechanisms, elucidated for a small number of species, depend on genotype and developmental stage. Remarkably, key enzymes and transporters involved in the main sugars-conversion and transport pathways have received considerable attention. In stone fruit trees, the presence of an elevated number of fruits alters the source-sink balance, with a consequent intensification of competition among them and between vegetative and reproductive growth. The main environmental factors affecting this balance and the agronomical/artificial manipulations of source-sink relationships to achieve adequate fruit production and quality are reviewed.

## Introduction

Growth and development in tree crops are combined processes in which the metabolic need of non-photosynthetic “sink” tissues, such as fruits, is balanced by the primary assimilation of photosynthetically active “source” tissues, such as mature leaves. The comprehension of source-sink relationships and the control of carbon partitioning among sinks in plants are very important to advance the knowledge about tree crops and the impact of yield-limiting factors (Pavel and DeJong, 1993). In fact, within a tree, fruits, which are strong sinks, compete for assimilates with each other, especially in case of high crop loads, as well as with vegetative organs, such as shoots, leaves, and roots (Pavel and DeJong, 1993; Ludewig and Flügge, 2013). For this reason, balance preservation between vegetative and generative growth, also achieved by the artificial manipulation of source-sink relationships, can be essential to ensure acceptable fruit production and quality (Fischer et al., 2012). On the other hand, fruit size is affected by the ratio between source organs (leaves) that provide sugars for growth and the number of sinks, such as fruit and other non-photosynthetic organs that compete for these (Grossman and DeJong, 1994). The partitioning of assimilates in economically important sink organs, such as fruits, is ruled by several processes, including photosynthetic rate, phloem loading, translocation throughout the phloem, phloem unloading, and uptake and metabolism of carbohydrates in sink organs (Patrick, 1997; Liesche and Patrick, 2017).

## Assimilates Production and Phloem Loading

Sugars are produced by fruit photosynthesis; nevertheless, over 90% of the assimilates required for peach growth are imported, and for cherries, this proportion is about 85% (Pavel and DeJong, 1993). Leaves are the most important structure for photosynthesis and assimilate production, even if a developing leaf can represent both a source and a sink (heterotrophic), by importing carbohydrates from other parts of the plant (Pavel and DeJong, 1993). Photosynthetic behavior of *Prunus* spp. has been widely described (Flore et al., 1996; Flore and Layne, 1999; Flore and Lakso, 2011), and authors found that values for the photosynthetic rate of these species range from 7 for almond to the highest 26.2 μmol m^−2^ s^−1^ of a plum variety, with cherries positioned really close to this value. However, some evidence has been reported for stone fruits indicating that leaf photosynthetic capacity varies greatly within the canopy, being affected by intrinsic factors, such as leaf age, exposition, and fruit amount (Testolin and Costa, 1991; Flore and Lakso, 2011). Moreover, Urban et al. (2003) have demonstrated that, in mango trees, the leaves in proximity to developing fruits showed increased photosynthetic capacity as compared to the others. Similar results have been reported in an earlier work carried out by Crews et al. (1975) showing higher rates of photosynthesis in peach leaves in close proximity to fruits; the same authors demonstrated that leaf photosynthesis peaked during the stage of maximum fruit growth rate. The removal of leaves (30%) in sour cherry (*Prunus cerasus*) decreased photosynthesis, but compensation, due to the rise of the photosynthesis rate in the remaining leaves, was observed when the amount of defoliation was reduced (Layne and Flore, 1992).

Little information about the leaf area index (LAI, ratio between leaf area and occupied soil area) is available for fruit tree species, although this parameter has a pivotal role in sunlight interception and therefore productivity. Peach canopies generally have a high LAI after the initial spring growth (6–8 m^2^ of leaf area per m^2^ of ground area; Fahey, 1992), but this value is highly variable depending on the species. Rajan et al. (2001), for example, carried out a study on 26 Indian mango varieties measuring LAI ranging from 1.18 to 4.48; authors demonstrated that the genotypes with a low LAI were better exposed to solar radiation and displayed higher-quality production.

Sugar transport, direction, and volume are determined by sink position and relative sink strength. Carbohydrates produced in leaf mesophyll are loaded into the phloem systems and unloaded in energy-demanding or storage tissues (sinks); both the mechanisms can be apoplastic (sugars cross the cell membrane) or symplastic (exclusively through the plasmodesmata-connected cells; De Schepper et al., 2013; Roch et al., 2019). Among the sugars synthesized in plants, only a small number of them, generally highly soluble and chemically inert, are transported in the phloem over a long-distance. Sucrose, which is less reactive than reducing sugars such as glucose and fructose, is the main form of carbon found in the phloem, but, in some species, polyols, such as sorbitol, are translocated in the phloem (Moing et al., 1997; Noiraud et al., 2001; Tyree and Zimmermann, 2002). Sorbitol has been detected in phloem sap of peach and apricot trees (Bieleski and Redgwell, 1985; Moing et al., 1997); stachyose and mannitol have been found in *Olea europea* L. (olive) and in coffee (Zimmermann and Ziegler, 1975; Flora and Madore, 1993).

**Figure 1:**
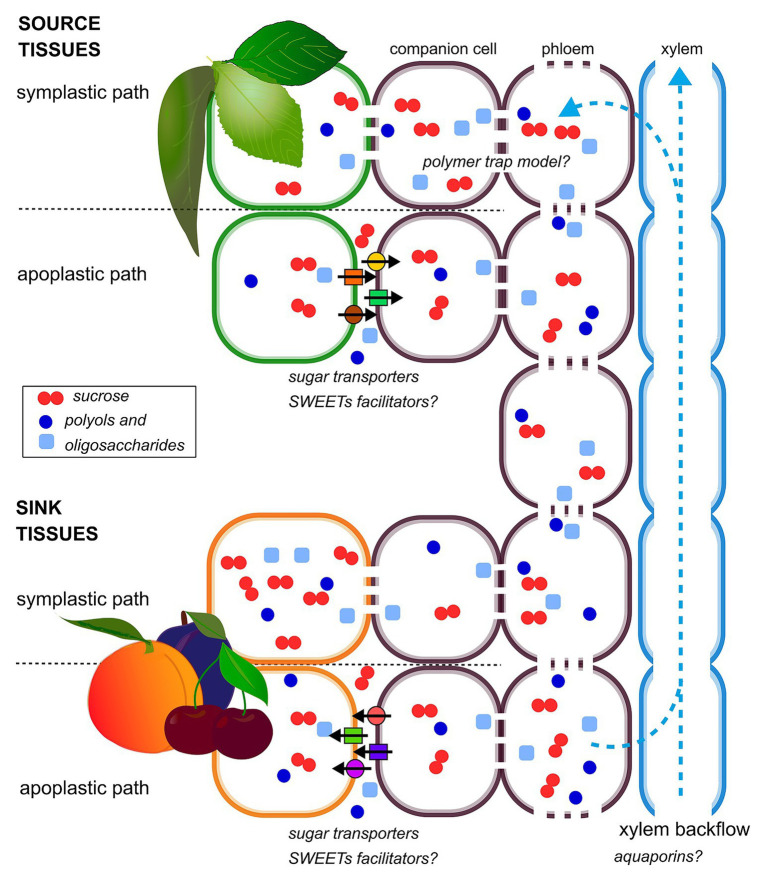
Schematic diagram of hypothetical phloem-loading and -unloading strategies in stone fruit plants. Sucrose, polyols, and oligosaccharides are indicated as a representative of the different sugar species transported and accumulated in stone fruit plants. Both apoplastic and symplastic paths are shown, in source and sink organs, according to their coexistence, spatio-temporally regulated.

In most of stone fruit species, the mechanisms of phloem loading have not been fully elucidated yet. The available data suggest that phloem loading can occur through an apoplastic or a symplastic path or possibly a combination of both, depending on the species and the sugar considered (Flora and Madore, 1993; Noiraud et al., 2001; Nadwodnik and Lohaus, 2008; Figure 1). The passive symplasmic mechanism has been identified in most fruit trees of Rosaceae family (Reidel et al., 2009; Fu et al., 2011; Roch et al., 2019). Despite the importance of sorbitol in this family, sorbitol transporters were found only in sink organs (Gao et al., 2003; Watari et al., 2004), making the knowledge on polyol phloem loading extremely limited. In plasma membrane vesicles, obtained from peach leaves, sucrose and sorbitol uptake exhibited saturated kinetics; these results, together with the observation of sucrose transporters in various plant tissues, suggest that sorbitol and sucrose loading is carrier mediated (Marquat et al., 1997; Nadwodnik and Lohaus, 2008).

There are different types of sugar transporter in plants, and these include sucrose uptake transporters (SUTs: sucrose/H+ symporters), hexose transporters (hexose/H+ symporters), and SWEETs (sucrose facilitator; Milne et al., 2018).

The functionality of a sucrose transporter (PpSUT1) has been recently demonstrated in peach. The pH-dependent activity of this transporter is consistent with the classification of other SUT1 as H+/sucrose symporters. Therefore, Zanon et al. (2015b) suggested that it mediates the phloem loading of sucrose in leaves, supporting the hypothesis of an apoplastic loading pathway in peach.

Conversely, phloem loading of oligosaccharides, such as raffinose and stachyose, is supposed to occur symplastically, and its mechanism has been described by the “polymer trap model” (Rennie and Turgeon, 2009). Briefly, sucrose produced in mesophyll of source leaves diffuses to companion cells *via* plasmodesmata, and it is utilized as a substrate for oligosaccharide synthesis which flows through the sieve tubes of the phloem.

## Translocation of Water and Solutes

The bulk of the water and solutes required for the growth of fruits and seeds is imported into them through the xylem and phloem present in their vasculature (Peoples et al., 1985; Pavel and DeJong, 1993). In the stone fruits, five vascular bundles (containing both xylem and phloem) enter the base of the fruit, then dividing to supply either the flesh or the seed. In the flesh, various branches diverge off the main bundles and ramify and anastomose throughout the flesh (Ragland, 1934; Tukey and Young, 1939; Sterling, 1953; Grimm et al., 2017). The relative contribution of xylem and phloem to fruit growth is dependent upon several factors that include the material that is being imported, the species of the stone fruit, the time of day, and both the tissue and the stage of fruit development (Matthews and Shackel, 2005; Morandi et al., 2007; Brüggenwirth and Knoche, 2016).

In sweet cherry, the input of liquid *via* the phloem is about 15% of the total imported before stage III, and it then increases to about 85–100%. Xylem flows (85% of the total liquid imported) greatly exceed phloem flows during stage II and then decrease during stage III to almost zero at harvest. Transpiration flow is similar to xylem stream during stage II, but greater than xylem flow during stage III (Brüggenwirth and Knoche, 2016). In prunes (*Prunus domestica*), liquid input *via* the xylem accounts for a large proportion of the total liquid imported during stage II; this proportion decreases during stage III and then increases again later in stage III (Matthews and Shackel, 2005). In Japanese plum, during stage III, input can occur *via* both the xylem and phloem, and the relative contributions are dependent on the time of day (Corelli Grappadelli et al., 2019). In peach, during both stages I and III, liquid inputs to the fruit *via* phloem (30% of the total imported) or the xylem (70%) are comparable. However, in both peach and sweet cherry, the relative inputs of liquid by phloem and xylem vary differently during the diurnal cycle (Morandi et al., 2007; Brüggenwirth and Knoche, 2016).

The movement of water and solutes from the phloem into the apoplast, and their subsequent uptake into sink cells, requires their passage across plasma membranes. Albeit diffusion can potentially contribute to such membrane transport, it appears that transporters and/or channels are needed, and these greatly increase the rate of transport (Milne et al., 2018). Although few studies on stone fruit aquaporins are present in literature, in a wide range of other plant species, it is established that these channel proteins have an important role in facilitating water exchange in fruits (Milne et al., 2018). Consistently, aquaporin encoding genes appear to be modulated both in peach fruit (Sugaya et al., 2001) and in cherry (Chen et al., 2019).

In addition, once the import of phloem water into sink cells exceeds the increase in their volume, surplus of water needs to be eliminated. In several fruits and seeds, this water is recycled back to the transpiration stream, and aquaporins are likely involved also in this process, allowing water to reach sink apoplast and then xylem (Milne et al., 2018). In peach, between 55 and 65% of the total water imported into peach fruits is lost by transpiration, and the remainder is used to increase the volume of the fruit, and no backflow *via* the xylem has been detected (Morandi et al., 2007). In sweet cherry, small xylem backflows occasionally occur around midday during stage III (Brüggenwirth and Knoche, 2016). In ripening Japanese plum, some xylem backflow occurs during the morning (Corelli Grappadelli et al., 2019). The low or non-existent backflows in peach and cherry are significant because they exclude the possibility that excess water is recycled back to the axial transpiration stream, as proposed in grape (Zhang and Keller, 2017).

The concentration of sugars in the xylem of stone fruits is low after bud burst (Loescher et al., 1990; Andersen et al., 1995b). For example, their content in cherry drops from 15 to 2–3 mg ml^−1^ xylem sap after bud break (Loescher et al., 1990). Similarly, the sugar concentrations in the xylem of the terminal shoots of both peach and Japanese plum (both growing in Florida, United States in early August) were 0.1–0.4 mM (Andersen et al., 1995a,b). By contrast, the concentration of sugars in the phloem is much higher; in peach, the aphid stylet technique allowed to detect values of 800 mM in leaf phloem (Nadwodnik and Lohaus, 2008) and 700 mM in phloem of the shoot apex (Moing et al., 1997). These concentrations of sugar in the phloem sap are consistent with values of 160 (peach) and 180 (sweet cherry) mg dry matter ml^−1^ phloem sap that were estimated by comparing the phloem flow into the fruit with the rate of dry matter accumulation by the fruit (Morandi et al., 2007; Brüggenwirth and Knoche, 2016). Further, the content of dry matter in the phloem of both peach and sweet cherry fruits changes little during fruit development (Morandi et al., 2007; Brüggenwirth and Knoche, 2016). The bulk of the sugar contents of the phloem of both the leaf and shoot apex of peach consists of sorbitol and sucrose, with a ratio of sorbitol to sucrose of 2.8–4.5 (Moing et al., 1997; Nadwodnik and Lohaus, 2008).

## Phloem Unloading

Water and solutes that are unloaded from the phloem can potentially move through the flesh either *via* the extracellular space (apoplastic route), directly from cell to cell by plasmodesmata (symplastic route) or possibly both (Nie et al., 2010; Zhang et al., 2014; Zanon et al., 2015b). In stone fruits, phloem unloading mechanisms have been clarified for a small number of species, showing that both symplastic and apoplastic mechanisms can occur, depending on developmental stage and genotype (Figure 1). In cherry fruit, an apoplastic step in sucrose and sorbitol unloading, involving sugar transporters has been shown (Gao et al., 2003). A similar behavior has been observed in jujube fruit (*Zizyphus jujuba*), but in this case, the predominantly apoplastic mechanism is interrupted by a transient symplastic unloading in the central stage of fruit growth (Nie et al., 2010).

In peach flesh, a fluorescent symplastic tracer [6(5)-carboxyfluorescein diacetate, CFDA] remained restricted to phloem strands during either stage I or at the beginning of stage III when the flesh was rapidly expanding, indicating a symplastic discontinuity between veins and parenchyma cells and suggesting that the apoplastic route predominates at these times (Zanon et al., 2015b).

A recent work in the ripening fruits of Japanese plum has shown that rapid switches between the apoplastic and symplastic routes can occur over the course of each day (Corelli Grappadelli et al., 2019). The high hydrostatic pressure of the phloem is thought to contribute to the symplastic movement of materials unloaded from the phloem (Morandi et al., 2010; Patrick et al., 2013); nevertheless, it has been recently shown that the hydrostatic pressure of the phloem from which unloading occurs could potentially be low (Milne et al., 2018). In peach flesh, xylem liquid, together with solutes and water that are unloaded from the phloem into the apoplast, appear to be largely transported through the flesh by the bulk flow of water (Morandi et al., 2007, 2010).

Subcellular compartmentation is crucial for sugar accumulation, and it can significantly affect metabolite concentrations (Patrick et al., 2013; Génard et al., 2014). Many attempts to elucidate the mechanism of carbohydrates uptake and sugar transporters have been performed in peach fruits. A carrier-mediated transport has been firstly hypothesized for mesocarp of young peaches (Vizzotto et al., 1996). More recently, the functional characterization of three peach sucrose transporters allowed proposing different roles for these proteins in sucrose distribution. In detail, PpSUT4, localized at the tonoplast in mesocarp tissue, should act in regulating sucrose efflux from the vacuole compartment, although a different localization for this transporter cannot be excluded (Zanon et al., 2015a). In peach flesh, two SWEETs are expressed, and it can be hypothesized that they might play a role in sucrose unloading from the phloem (Zanon et al., 2015b). However, consistently with the importance of sorbitol in peach fruit, several transcripts have been annotated as *Prunus persica* sorbitol transporters, and they are more numerous than their orthologues (Verde et al., 2013).

Key enzymes involved in the main sugar conversion pathways, such as sucrose synthases and invertases, have gained attention for their involvement in determining sink strength during fruit development, and therefore, supporting the process of unloading, by accentuating the sucrose gradient between phloem and the parenchyma cells (Koch, 2004; Nonis et al., 2007; Morandi et al., 2008). Hubbard et al. (1991) indicated that activities of the sucrose metabolizing enzymes, including sucrose phosphate synthase, in fruit such as peach and mango, are pivotal in defining the content of soluble sugar in fruits. However, the relationships between enzyme activities and their products are often non-linear and are hard to evaluate; for this reason, a new kinetic model of sugar metabolism has been proposed in peach. The model suggests a different utilization of sucrose and sorbitol from sap: almost all sucrose, which is not hydrolyzed into the apoplasm, would be stored in the vacuoles; on the other hand, sorbitol would be massively catabolized in the cytosol, representing the main driver for the respiration and the synthesis of structural compounds (Desnoues et al., 2018). However, this model has recently been questioned and it has been suggested that sucrose could provide an appreciable proportion of the substrate utilized by the metabolism of peach flesh (Walker et al., 2020). Carbon translocation pathway, from leaves to fruits, has been widely studied in *Prunus* spp. (Kappes and Flore, 1986; Flore and Layne, 1999). In cherry and peach, during stage II of fruit growth, all leaves supply new shoots and fruit growth at the same time; during stages I and II, the leaves closest to fruit export basipetally, and fruits attract assimilates more strongly (Kappes and Flore, 1986). Interestingly, in sweet cherry, in which fruit matures even faster than peach and apricot, carbon labeling experiments displayed that fruit adjacent to the carbon source were very strong sinks if compared with shoots (Ayala and Lang, 2008).

The main sugars accumulated in stone fruits include fructose, glucose, and sucrose, along with other minor saccharide containing stachyose, sorbitol, raffinose, rhamnose, arabinose, galactose, and xylose (Gross and Sams, 1984; Serrano et al., 2005; Cantín et al., 2009). Both sorbitol and sucrose are imported in peaches and other stone fruits (Hansen and Ryugo, 1979; Gao et al., 2003; Lo Bianco, 2009). However, in peach, some authors have hypothesized a preferential consumption of sorbitol in vegetative sink tissues, such as young leaves and cambium, close to the source (Moing et al., 1992; Lo Bianco et al., 1999), and, conversely, a key role of sucrose in fruit growth (Vizzotto et al., 1996; Zanon et al., 2015a,b).

## Competition Among Sinks

In plants, several sinks compete for the available photoassimilates. This generates a priority system among them, in which, during the early developmental stages, roots and young leaves are the main sinks, whereas, during the reproductive stages, fruit and seeds become priorities (Lemoine et al., 2013). Partitioning of carbohydrates within a tree is not a genetically programmed process, but it is the result of a combination of competing organs and their relative aptitudes to compete for assimilates (Lakso and Flore, 2003). Fruits attract photosynthates and thus increase the assimilate production of leaves; on the other hand, the deficiency of fruits in the canopy cause accumulation of carbohydrates in leaves (Fischer et al., 2012). However, a study on sour cherry demonstrated that the sink effect on photosynthesis is not obvious under all circumstances, and this can be ascribed to the masking effect of competing sinks, of source-limiting conditions, or to the environmental conditions which favor assimilates production (Flore and Layne, 1999).

The potential capacity of a fruit to accumulate assimilates (sink strength) depends on its size, its location, and distance from the source (Pavel and DeJong, 1993). Notwithstanding, in stone fruit trees, the presence of an elevated number of fruits alters the source-sink balance, by increasing assimilate accumulation into the fruits and/or by inducing an intensification of competition among them and between vegetative and reproductive growth (Morandi et al., 2008; Costa et al., 2018). This is also the case in olive (Rosati et al., 2017, 2018b,c). As a rule, high crop loads cause a decrease in tree growth (Intrigliolo and Castel, 2009), mainly during the last phases of fruit development (Intrigliolo et al., 2014). In addition, high crop load induces a condition of constrained carbohydrates supply, which amplifies competition among fruits and causes low fruit quality at harvest (Quilot and Génard, 2008; Abrisqueta et al., 2017). In olive, which is a drupe producing species, fruit production proportionally reduced shoot length, but the leaf biomass-to-shoot wood biomass ratio increased and this may be viewed as a plant strategy to better support fruit growth in high fruit load years, also given the greater and earlier ability of short shoots to export carbon (Rosati et al., 2018a). In other words, when there is a high fruit load, the vegetative growth is reduced, but there is a higher proportion of leaf biomass with respect to the wood biomass in new short shoots.

Shoot apices can also represent competitors for a growing fruit (Génard et al., 1998a). This observation can provide an explanation for the poor quality of fruits located in the terminal section of axes. Particularly, during the earlier phases of fruit growth, when cell division primarily occurs, shoot apex has been demonstrated to be the strongest sink both in peach and Japanese apricot (Corelli Grappadelli et al., 1996; Tsuchida et al., 2011). A recent research, carried out in different plum varieties, has pointed out the important role played by the mesocarp cell number in establishing fruit sink strength (Cerri et al., 2019) and confirmed observations obtained in other species such as peach and Japanese apricot (Yamaguchi et al., 2002, 2004). In early ripening peach cultivars, the phase of cell division in fruit overlaps with that of intensive shoot growth, thus reducing fruit fresh weight (DeJong et al., 1987). On the contrary, in sweet cherry, fruits located near source leaf appear to have stronger sinks as compared with growing shoots, the effect being also modulated by leaf:fruit ratio (Ayala and Lang, 2008). As a rule, competition between vegetative and reproductive activity may be favorably handled in cultivars characterized by a clear separation between the two stages, as observed in different peach varieties (Ruiz-Sanchez et al., 2010).

## Environmental Factors and Management Practices Influencing the Production of Stone Fruit Trees

### Agronomical Factors

#### Thinning

A number of manipulations have been proposed to modulate the competition inside trees and to maximize commercial yield. In fact, even if fruit trees show fruit load self-regulatory systems, linked to the partitioning of assimilates to different organs (Dennis et al., 1983), these are frequently insufficient to obtain good fruit marketable size and quality at harvest. The balance among different competing organs is performed by fruit or flower thinning and is annually carried out on peach and nectarine, while occasionally on apricot, plum, and, in recent years, also on cherry (Costa et al., 2018). In different cultivars of sweet cherry, thinning induced an increase in fruit fresh weight through a positive effect on cell length (Olmstead et al., 2007); while, in other species, the response to this agronomical technique is controversial.

Thinning could affect competition for assimilates at a different level; in fact, reducing crop load leads to the increase of the sink strength of remaining fruits and affecting endogenous factors, such as hormonal and carbon balance, regulates the transition of buds to reproductive phase, thus determining the potential production of the following season (Costa et al., 2018).

Fruit thinning is primarily adopted for peach, and the most promising approach seems to be the “multiple application strategy,” which allows monitoring the crop load at different phenological stages and implicates the application of chemicals and/or agronomical techniques to reduce fruit load (Greene and Costa, 2013). However, thinner response is affected by several internal and external parameters (i.e., genotype, environmental conditions; Costa et al., 2006). Therefore, thinning strategies may be fine-tuned, modulating thinning intensity in different part of the canopy and reducing the presence of fruits in the distal portion of 1-year old shoot and in the internal part of the tree. An early thinning, after an adequate fruit set, could regulate fruit fresh weight in apricot, possibly affecting the mechanism of assimilate transport between sources and sinks (Stanley, 2016).

Several studies investigating changes in fruit growth, total soluble solids (TSSs), pulp:stone ratio, fruit quality, and fruit diameter, along with the effects of leaf:fruit ratio were carried out. Thinning on mango “Lirfa” displayed the best results (highest fresh weight) when the leaf/fruit ratio was approximately 100 (Léchaudel et al., 2004). Other authors observed that thinning (optimal 40–50 leaves/fruit) positively affected sugar content and pulp:stone ratio in the “Rubidoux” peach (Fischer et al., 2012). On the other hand, a study on the effects of source-sink balance on quality parameters of two nectarine cultivars with different harvest time demonstrated that thinning induces a transient accumulation of soluble sugar in leaves, resulting in reduced photosynthesis and stomatal closure with no significant impact on final fruit size. Authors ascribe these controversial results to the influence of crop load on fruit-water relationships, possibly improved by the frequent irrigation in unthinned plants, allowing the fruit to reach its maximum potential (Andrade et al., 2019).

Webster and Spencer (2000) reviewed the strategies for crop load reduction to improve fruit quality in plum and apricot trees; they suggest that manual thinning is still the most precise and reliable method for these species. Authors also concluded that a better understanding of the climatic site, tree, and management factors influencing seasonal variations in fruit set and abscission is needed. This knowledge could be helpful to predict fruit set and abscission, which, in turn, would aid the grower in decisions on crop load adjustments.

An accurate trial recently demonstrated that fruit quality of the European plums is almost unaffected by either mechanical or chemical thinning, probably due to the utilization of an ethylene releasing compound. On the other hand, mechanical blossom thinning has been proposed to overcome or avoid alternate/biennial bearing (Seehuber et al., 2011). Interestingly, the same authors observed that stone fruit, as compared to pome fruit, mature within a shorter time, and a greater number of leaves are required for the same final fruit size. In addition, a lower number of fruits need to be removed to obtain a faster growth and a sugar increase in stone fruit, and an upper saturation threshold is rapidly reached without further effects.

#### Cultivar and Rootstock

Breeding programs have given rise to many stone fruit cultivars, aiming to meet several objectives, such as fruit quality, resistance to biotic and abiotic stresses, and the extension of the harvest season with early and late varieties. Source-sink relationships differ among early-, mid-, and late-ripening varieties, as evidenced, for example, by the diverse nutrient content of peach fruits. One of the main reasons is related to the shorter period of competition between leaves and fruits in earlier ripening cultivars. In fact, during the postharvest phase, trees are allowed to store nutrients in the permanent structures to sustain the first stages of growth in the following season (Zhou and Melgar, 2019).

In several stone fruit trees, rootstocks have been proposed to play a regulative role in the interaction with environmental conditions, and also in determining fruit quality (Caruso et al., 1996; Albás et al., 2004; Sitarek et al., 2005; Daza et al., 2008; Ruiz and Egea, 2008; Iglesias et al., 2019), through a regulation of photosynthesis, water relations, tree vigor, and diverse reproductive traits (Zarrouk et al., 2005; Goncalves et al., 2006; Basile et al., 2007). Interacting with temperature and soil, rootstocks of different stone fruit trees regulate yield, fruit weight, and nutrient partitioning to the fruits (Layne, 1994; Rato et al., 2008). The effect on tree vigor appears to influence fruit quality both directly, modifying the competition between vegetative and reproductive activity, and indirectly, determining light interception/shading by the canopy (George et al., 2005; Font i Forcada et al., 2012; Gullo et al., 2014). Commonly, there is a tight relationship between bearing capacity and canopy size (Westwood, 1978). Rootstocks have also a role in the exchange of endogenous signals (such as hormones) with scion, and among the different plant organs, modifying the source-sink relationships (Tombesi et al., 2010; Minas et al., 2018).

In cherry, the choice of the right rootstock continues to be a topic of great interest due to its importance for productivity (Correia et al., 2017). Rootstocks have noteworthy aptitudes of adaptation to different growing conditions, but a significant effect of the rootstock on fruit quality has been demonstrated by different authors for both sweet cherry and peach (Giorgi et al., 2005; Usenik et al., 2010; Orazem et al., 2011). Several studies have investigated the influence of the scion/rootstock combination on cherry fruit quality (Whiting et al., 2005; Goncalves et al., 2006; Correia et al., 2017; Morandi et al., 2019), showing that water relations and photosynthesis of cherry trees are largely influenced by the rootstock genotype. Recently, the significant effect of different size-controlling rootstocks has been remarked; a semi-dwarfing (Gisela™6) rootstock, for example, allowed a higher productivity and fruit sugar content, thanks to the fruit increased accumulation of osmotic compounds and competitiveness toward shoots (Morandi et al., 2019). Moreover, fruit firmness varies according to scion and rootstock combination; “Burlat” cherries, for instance, decrease their firmness when grafted on CAB 11E (semi-vigorous rootstock) but they are firmer when grafted on Gisela 5 (dwarfing rootstock; Goncalves et al., 2006).

On the other hand, in peach, rootstocks of similar vigor can produce fruit of different quality, indicating that vigor is not the only parameter that affects fruit production, but the different genetic origin can play an crucial role in determining yield quality (Giorgi et al., 2005; Usenik et al., 2010; Orazem et al., 2011).

Orazem et al. (2011) investigated the effect of seven rootstocks grafted with Royal Glory and Redhaven peach cultivars on tree vigor, yield, and fruit quality. Authors showed that the degree to which rootstocks affect fruit weight, sugars, phenolics, and organic acids content levels varies from cultivar to cultivar.

These outcomes point out that the effect of rootstock is significantly intricate and cannot be measured by vigor alone. Therefore, field performance of the rootstock is still the main parameter for its choice, and its impact on fruit quality should not be disregarded.

#### Pruning

Winter and summer pruning are largely applied techniques in all fruit trees, including stone fruit trees, providing a valuable method for size control.

Several studies about the effect of summer pruning on carbohydrate content in peach and cherry have been carried out and report similar findings (Clair-Maczulajtys et al., 1994; Ikinci, 2014). Ikinci (2014) suggested summer pruning as a standard cultural technique in the management of peach trees. The study pointed out that this practice reduced shoot length, stimulated shoot diameter enlargement, decreased fruit yield, and increased fruit weight; in addition, this technique, if applied each year, increased significantly fruit soluble solids content (SSC). The increase in fruit size and quality has been attributed to higher photosynthate availability in the fruit of summer-pruned trees due both to the increment in photosynthetic photon flux density (PPFD) and to the elimination of competing sinks, i.e., watersprouts. The improved light exposure, in turn, may increase fruit sink activity, thus positively affecting fruit size, as shown for nectarines (Day et al., 1989). On the other hand, competition between vegetative and reproductive growth, that influences fruit abscission, is well-known for deciduous fruit trees. McFadyen et al. (2011) suggested that pruning in macadamia increases fruit drop and reduces yield, as the result of the combined negative effects of leaf removal and competition on carbohydrate availability from new shoots growth. Authors demonstrated that pruning determined an increase in fruit abscission and this effect was local and related to the aforementioned competition for carbohydrates.

It is also noteworthy that, in apricot, the position along the shoot, fruit number, and fruiting node leaf area have great importance on fruit quality; the higher FW and SSC are measured in fruits present on distal zones of 2-year-old wood and the lower in fruit from distal zones of 1-year-old wood. These observations suggest that the source-sink ratio should not be considered at the tree level, as diverse positions into the canopy can differentially regulate growth, for example, interacting with light or type of bearing structure. On the other side, results allow to develop more targeted orchard practices such as pruning or training system, favoring the presence of more efficient bearing structures, depending on the species, e.g., older wood (2–3 years) in apricot (Stanley, 2016).

#### Pre-harvest Fruit Bagging

Pre-harvest fruit bagging is commonly used as an effective approach in Japan, Australia, and China. Bagging is a physical protection technique, applied to different fruits, aiming to improve their appearance by promoting skin coloration and reducing the incidence of fruit cracking, but can also change the microenvironment for fruit development (Sharma et al., 2014). Pre-harvest bagging on peaches and nectarines has been investigated by several authors (Li et al., 2001; Zhang et al., 2015). Bagging modified SSC content, with a different trend, depending on the type of bag and species (Sharma et al., 2014). However, this could not be ascribed to the reduced light intensity but rather to the micro-environment modification that affected the rates of transpiration and respiration in fruits (Zhang et al., 2015).

#### Fertilization

It is known that plant nutrition could affect different traits of fruit quality such as fruit appearance, texture, and taste. Moreover, postharvest fruit properties, and mainly storage life length, appear to be influenced by fertilization, which has a role in susceptibility of fruits to mechanical damage, physiological disorders, and decay (Cuquel et al., 2011). Nitrogen (N) and potassium (K) are among the most important nutrients needed by plants. However, an excess of N fertilization could negatively impact the stone fruits quality by diminishing flesh firmness and sweetness, decreasing red color development, and increasing susceptibility to postharvest diseases (Crisosto et al., 1997; Rettke et al., 2006; Cuquel et al., 2011).

Most of the effects of nutrient deficiency/supply on stone fruit quality and sugar content are indirect and they depend on the nutrient impact on the canopy growth and on the source-sink balance, which is species-specific and stage-specific. In sweet cherry, nitrogen fertilization favored the carbon allocation into fruits and fine-roots while hampering the vegetative growth (Artacho and Bonomelli, 2017).

In peach, even though flower density and fruit set are generally not affected by N, overall yields are decreased by N deficiency because of reduced fruit size and less fruiting sites due to shorter shoot (Layne and Bassi, 2008). Diversely, in low chill peach cultivars, the reduction of fruit quality after high N fertilization has been related to an increase of vegetative growth and, as a consequence, of fruit shading, or to an increase of competition for carbohydrates between fruits and shoots in a critical period (Wert et al., 2009).

However, considering that N is stored and recycled each year to sustain new growth, it is important to address peach orchard fertilization based on tree N status. It has been demonstrated that the increment in N supply over the optimum level for tree maintenance does not increase yield. Yet, a recent study suggests that lower N rates may advance fruit maturation by increasing color and soluble solids (Rubio Ames et al., 2020).

There is some controversy over the effect of K on fruit quality; in detail, no correlation between leaf K content and fruit soluble solids amount has been shown in peach (Layne and Bassi, 2008). However, Tagliavini and Marangoni (2002) provided evidence that fruit size, SSC, and color can all be improved when peach trees are not deficient in K (1.35–1.6% K in leaf).

Peach is much less sensitive to boron (B) deficiency than most other plants, and this is possibly due to the easily translocated compound that is formed from B and sorbitol (or other sugar alcohols) that accounts for the high mobility of this element (Hu et al., 1997; Layne and Bassi, 2008).

In apricot (cv. Canino), different levels of N, phosphorus (P), and K fertilizers could affect biochemical markers implicated in fruit quality. The authors pointed out that the mode of N distribution could have a major role in the quality of apricot fruits, but with negligible effects on sugar content. In addition, the N-K balance was found to be the most important parameter to increase both sugars and phenolic compounds accumulation (Radi et al., 2003).

### Environmental Factors

#### Light and Temperature

Environmental and soil conditions interact with endogenous factors and management practices to set the response of the tree and the productive performance. For instance, unsuitable winter temperature may only partially fulfill the chilling requirement, with negative consequences on regular flower formation (Petri and Leite, 2004; Blanke and Kunz, 2009) and reproductive activity. In peach, it has been demonstrated that different environmental factors (e.g., temperature, light, and precipitation) and field practices (such as irrigation, rootstocks, pruning and training system, and thinning) could affect the assimilate partitioning into the fruits, by changing both plant photosynthetic source efficacy and, therefore, carbohydrates availability and individual sink strength (Cirilli et al., 2016). A number of models were developed trying to estimate the effect of several factors on source-sink relationships and to improve efficiency in peach and other fruit crops (Quilot et al., 2004; Lescourret and Genard, 2005; Sonnewald and Fernie, 2018). Besides the number of fruits present on the tree and the fruit:shoot balance, also the relative position on the canopy of source and sink organs may influence the movement of resources (Génard et al., 1998b). In apricot, the higher SSC content of fruit was related both to light exposure and the type of bearing structure (Dichio et al., 1999; Lichou et al., 1999; Stanley et al., 2014). Longer shoots show lower fruit set as compared with spurs and maybe as a consequence, in the latter, of higher starch content in ovaries and ovules (Julian et al., 2010), earlier flower initiation during previous season (Sakayarote et al., 2005), and lower chilling requirement (Austin et al., 1992).

Light availability influences fruit development directly through primary carbon metabolism, and indirectly, favoring the translocation of assimilates from reserves accumulated in more exposed parts of the trees or by increasing fruit temperature, which enhances its sink activity (Génard and Bruchou, 1993; Cherbiy-Hoffmann et al., 2013; Reale et al., 2019).

The sunlight use efficiency has long been investigated in order to improve fruit quality at harvest. An optimum photosynthetically active radiation (PAR) interception of orchard may be achieved considering different cultural choices as orchard design, training system, and pruning that, as a whole, may also improve PAR distribution into the canopy (Jackson, 1980; Corelli Grappadelli and Lakso, 2007; Reale et al., 2019).

The interaction between light effect and canopy position has been pointed out in peach, where fruits with poor quality are usually located on older wood in the lower and central part of the tree (Luchsinger et al., 2002). This relationship was also confirmed by artificial shading that, especially if applied during the final phases of fruit growth, when assimilated accumulation is high, induces a reduction of SSC (George et al., 1996). Therefore, peach appears to be particularly sensitive to light exposure, especially in stimulating soluble sugars accumulation during the final phases of fruit growth (3 weeks) and in determining fruit quality (Marini et al., 1991; Gullo et al., 2014). Reale et al. (2019) recently demonstrated that the effect of light availability on olive fruit development is cultivar-specific, and the greater sensitivity was related to a delay in the endocarp lignification.

The exposure of fruit to sunlight also induces an increase of fruit temperature, and through an effect on respiration and transpiration rates, of sink strength (Marini et al., 1991; Pavel and DeJong, 1993; Génard and Baret, 1994). However, excessive temperature, especially during the earlier phases of growth, may induce a reduction of fruit developmental period and SSC accumulation (Lopresti et al., 2016).

#### Soil Conditions

Among factors that have a decisive impact on orchard production and that can be controlled are the level of macro‐ and micro-elements in soil. The influence of fertilizer’s application, fruit species and cultivars, rootstock, and its vigor on plant nutrition has been pointed out in the previous sections. Similarly, mineral nutrient status can affect assimilate partitioning either directly by modulating phloem loading and transport or indirectly by depressing sink demand (Marschner et al., 1996). On the other hand, plants deficient in macroelements improve their ability to acquire these nutrients by altering their carbon partitioning to favor root growth (Hermans et al., 2006).

Soil conditions, such as water content, soil type and structure, organic matter content, and soil pH, affect individually the availability of nutrients, but also biological soil properties are pivotal. In general, macroelements are available to the root of fruit tree in a wider range of soil pH; in contrast, the availability of micronutrients relates closely to soil pH. The positive effect of soil organic matter content on availability and assimilation of nutrients has long been known. Naturally, organic matter or organic fertilizers, when applied to the soil in the fruit orchards, may be mineralized over time by soil microorganisms, which would increase the total organic C content and the metabolic activity of the microorganisms (Milošević and Milošević, 2020). Beside their role in mineralizing organic nutrients for fruit tree growth and development, microorganisms such as symbiont fungi (i.e., arbuscular mycorrhizal fungi) can increase plant’s nutrient uptake, especially P, but also plant’s tolerance to drought conditions, salinity, and biotic stresses, as reported for peach and peach-almond hybrids (Calvet et al., 2004), with indirect beneficial effects on the fruit quality (Ortas, 2018).

#### Water Availability

Another important environmental factor influencing physiological and metabolic activity in plants is water availability, and deficit irrigation (DI) has been adopted in several stone fruit species to regulate competition between vegetative and reproductive growth (Pérez-Pastor et al., 2007; Ruiz-Sanchez et al., 2010; Lopresti et al., 2014). In peach and nectarine, a multi-year research pointed out that DI favors carbon partitioning to fruits as compared to vegetative growth, and positively affects fruit quality, although the influence is also related to other factors such as stress intensity and period of application, crop load and thinning, and cultivar precocity (Thakur and Singh, 2013; Falagán et al., 2015; De la Rosa et al., 2016). Particularly, in extra-early nectarine trees, the postharvest phase represents a non-critical period for fruit to apply DI, even if during this stage floral differentiation occurs and assimilates are mainly accumulated to sustain the following season’s early growth (Handley and Johnson, 2000). Moreover, postharvest DI reduces flower number in the subsequent year without affecting fruit quality, conceivably by a reduction of tree size (Girona et al., 2005). In early nectarines, DI treatments induce a reduction of tree size, therefore suggesting to adapt the thinning strategy to this issue (Vera et al., 2013).

In apricot, water stress imposed after harvest did not significantly affect tree yield of the following year, but improved fruit quality. However, when DI was applied during the stages II and III of fruit development, a lower production was obtained, due to a reduction of fruit size and vegetative growth, respectively (Pérez-Pastor et al., 2007, 2009). On the other hand, almond yield appears almost unaffected by moderate water stress, even when applied during kernel development, possibly as a result of intense sink strength exhibited by the fruits during this phase (Lipan et al., 2019).

Stone fruit trees with higher crop load showed an higher susceptibility to water stress, which maybe due to a reduced partitioning of carbon toward above ground organs and, therefore, to an impaired root growth (Berman and DeJong, 1996; Lopez et al., 2007; Intrigliolo et al., 2013). Therefore, DI and thinning could be efficaciously adopted to regulate source-sink relationships into the trees, allowing, in different genotypes and environmental conditions, the achievement of optimal fruit production and quality (Embree, 2007; Intrigliolo and Castel, 2010; Intrigliolo et al., 2013).

## Concluding Remarks

Environmental factors exert a strong control on the timing of plant phenology, and understanding their effects is an essential step to allow predictions about plant responses, to optimize source-sink relationships in crops, and to target orchard management practices.

In the recent decades, the rate of climate changes increased dramatically, especially as regards average temperature, and this has triggered mechanisms of plant adaptation to the modifying environmental conditions. One of the main determinants of global warming is considered to be the concentration of CO_2_ in atmosphere, which is rapidly increasing, and is considered to be reached in 2017, a level of 405 ppm (Dlugokencky et al., 2018). In the short term, these changes have a positive impact on plant photosynthesis, and on sucrose production into the leaves, and depending on the species, plants rearrange their source-sink relationships (Makino and Tadahiko, 1999), mainly by reducing the root:shoot ratio (Farrar and Williams, 1991). However, in the long term, the increase in leaf sucrose concentration can activate a negative feedback on photosynthesis, with a detrimental effect on the productivity (Lemoine et al., 2013). Therefore, the new scenarios have to be taken into account to elucidate the mechanisms adopted by the plants to cope with these changes and to develop strategies to improve yield and fruit quality.

## Author Contributions

All authors have contributed significantly to the work and approved it for publication.

### Conflict of Interest

The authors declare that the research was conducted in the absence of any commercial or financial relationships that could be construed as a potential conflict of interest.
